# Laparoscopic Management of Large Right Paratubal Cyst: A Case Report

**DOI:** 10.31729/jnma.4982

**Published:** 2020-07-31

**Authors:** Rakina Bhansakarya, Shanti Subedi

**Affiliations:** 1 Department of Obstetrics and Gynecology, Nobel Medical College and Teaching Hospital, Biratnagar, Nepal

**Keywords:** *adnexal mass*, *cystectomy*, *laparoscopy*

## Abstract

Paratubal cysts are generally small but there are rare cases of large paratubal cyst and this case is one of them. Here we report a case of a young female with complains of abdominal fullness since 3 months. On examination, a huge mass (25 × 25 cm) extending from symphysis pubis upto xiphisternum was noted. Ultrasongraphy showed a cystic mass of 27 × 27 cm. Intraoperatively, the cyst was paratubal. It was drained with the help of veress needle and laparoscopic cystectomy was done. A large adnexal cyst extending above umbilicus is traditionally managed by laparotomy. But with the advent of laparoscopy, even a huge cyst can be managed by laparoscopy.

## INTRODUCTION

Paratubal cyst are remnants of Mullerian/Wolffian/ mesothelial origin. These are incidental findings during ultrasonography or operation and are rarely symptomatic. Generally, they are less than 8 cm in size.^1^

Laparoscopy is the gold standard in the treatment of benign adnexal mass. But when the cyst is large then due to chance of malignancy and also space constraint, many surgeons prefer laparotomy over laparoscopy. Here we report a case of a huge paratubal cyst which was managed by laparoscopy.

## CASE REPORT

A 25-year-old female came with complain of abdominal fullness since 3-4 months and sensation of mass per abdomen since 12 days. She was unmarried with a regular menstrual cycle and was not sexually active. As per patient, she had joined weight loss classes for 2 months, during which she had a visible weight loss of her arms and thighs but the abdominal fullness did not decrease and only then she noticed a mass in her abdomen. She does not have any other complaints. She had no significant medical or surgical history.

On examination, general condition was fair, huge abdominopelvic mass of around 32 weeks size (25 × 25 cm) extending from the symphysis pubis to 5 cm below xiphisternum and occupying the whole abdomen. The mass was cystic with smooth surface, freely mobile and lower margin could be felt.

**Figure 1.**
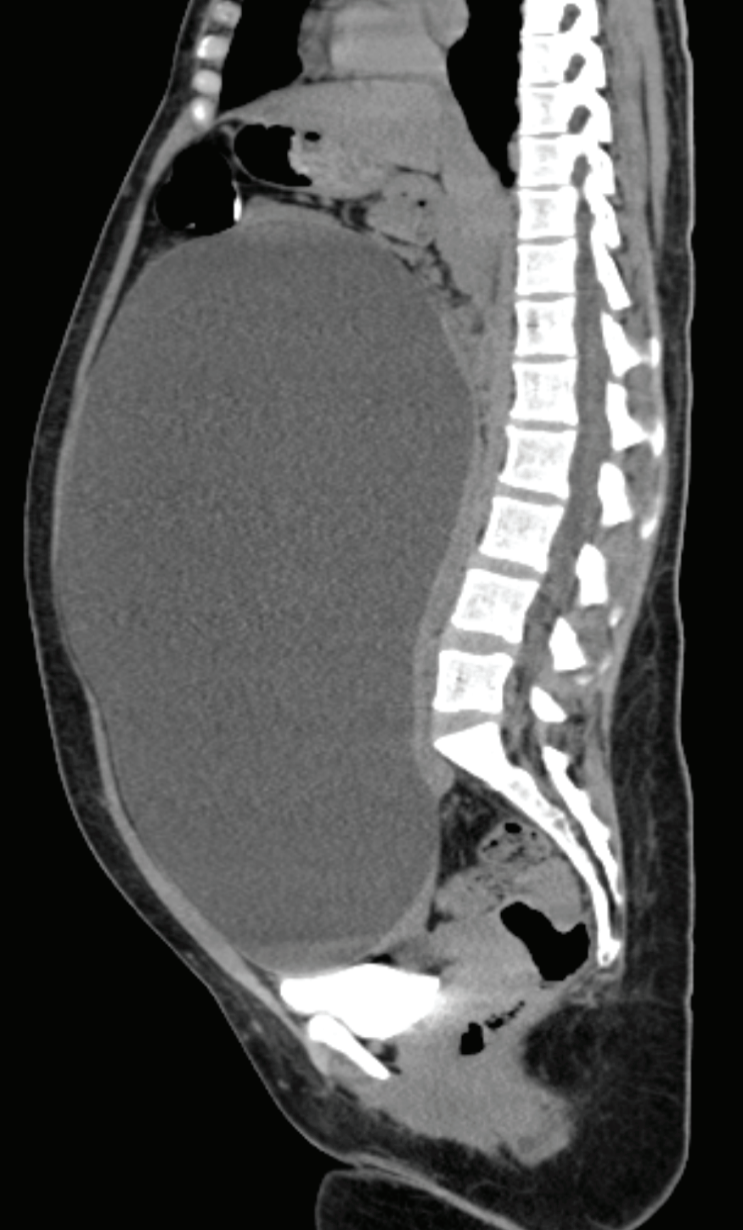
CT scan of cystic abdominopelvic mass.

Ultrasonography showed a cystic lesion of 27 × 27 cm in pelvis. Computed tomography (Figure 1) showed a cystic lesion of 28.8 × 25.8 × 15.4 cm with a thin wall, no septations, or solid components extending from pelvis upto epigastrium. Uterus was normal and bilateral ovaries not visualized in imaging studies.

Tumor markers-Cancer antigen-125 (CA-125): 29 IU/L, Carcinoembryonic antigen (CEA): 2.5 IU/l, Alpha feto protein (AFP): 2.85 IU/l, Lactate dehydrogenase (LDH): 174 IU/l were normal.

With the preop diagnosis of ovarian tumor, laparoscopic cystectomy was planned under general anesthesia.

Bowel preparation and antibiotic prophylaxis was given one day before operation theatre. Intraoperatively, there was challenge in inserting the primary trocar as the mass occupied the umbilical as well as left hypochondrium regions so drainage of cyst was done with veress needle and around 3 liters of serous fluid was drained. Primary trocar insertion in the palmer's point was done along with two 5 mm secondary port in right and left iliac fossa under vision. Pneumoperitoneum of 12-15 mmHg was created. A 30-degree scope was placed through by palmer's point.

**Figure 2.**
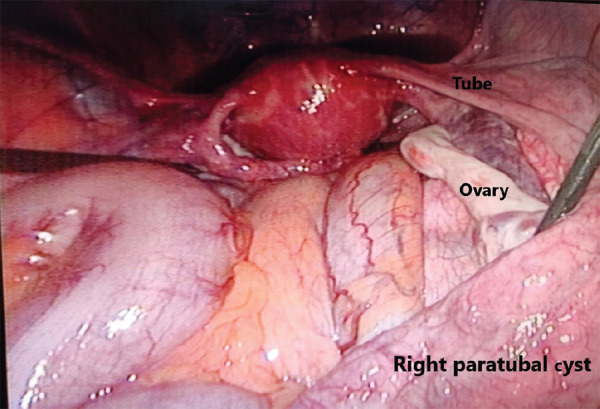
Laparoscopic picture of decompressed right paratubal cyst.

Intraoperatively, paratubal cyst was extending from the mesenteric border of the uterine surface upto the fimbria of the right tube, the cyst had glistening smooth surface and did not have any solid growth. The right tube was stretched over the mass and intact, right ovary was hypertrophied but normal looking. Uterus, left tube and left ovary were normal. There was no ascites, peritoneal, or omental growth (Figure 2).

Remaining 2 litre fluid from the cyst was aspirated. Cystectomy of right-sided paratubal cyst was done by dissection and counter traction. The right tube and ovary were preserved. There was no need for suture placement. Hemostasis was secured with cautery. Due to the large size of the cyst, there was difficulty in handling the mass intraoperatively and also in retrieving the specimen. For the retrieval of the mass, the incision in the right secondary port was extended and 10 mm port was created.

After removing the mass, peritoneal washing was done. Total time taken for the surgery was 120 minutes.

On the cut section, the cyst was 20 × 18 cm and had a smooth glistening surface without any solid mass, surface excrescences, or papillary projection. On the cut section, it was uniloculated cyst with a smooth inner surface. She had no post-operative complications and hospital stay was 48 hours.

Histopathology report showed cyst wall lined by intact cuboidal to low columnar epithelium with stratifications and ciliated cells at places. Subepithelium has fibrocollagenous tissue with lymphomononuclear infiltrate suggesting Serous cystadenoma. The patient had been followed up till 1 year and there were no recurrences.

## DISCUSSION

This case was special to us as it is the first time that we have performed laparoscopy for such a huge mass in our hospital. In our setting, mass of >24 weeks size is generally managed by laparotomy but taking into consideration, the young age of patient and benign characteristics noted in imaging and tumor markers, we decided to perform a laparoscopy to provide her treatment with least morbidity and minimal scar. The additional advantage was to lessen post-operative adhesions so that her chance of fertility is not hampered. Moreover, this is the largest paratubal cyst reported in literature in Nepal which was managed laparoscopically. In our case, we have decompressed the cyst by drainage of the cyst via veress needle, similar to that done by Nagle and Magos.^2^ The literature states many ways of managing such huge cyst-like open Hasson's technique for trocar insertion and laparoscopically aspirating the cyst as done by Thakur A.^3^ Ates O has inserted nephrostomy catheter in the cyst under ultrasonography guidance and aspirated the content of the cyst completely so that there is no intraperitoneal spillage of the cyst.^4^

The largest cyst managed laparoscopically was by Alobaid where he has removed an ovarian cyst measuring 42 cm in a 20-year-old female. In this case, the abdomen was opened by Hasson method and drainage of the cyst was done laparoscopically by suction and irrigation.^4^

Dolan in 2006 removed an ovarian cyst measuring 40 × 30 cm in 16 years old.^5^ In this case, the cyst was decompressed by a mini-laparotomy incision given in umbilicus and then extirpation of cyst was done laparoscopically.

The largest paraovarian cyst is reported by Letourneur in 19-year old which was 36 × 25 cm in size and was managed by laparoscopy and minimal suprapubic laparotomy.^6^

There are chances of upgrading of stage of cancer due to spillage during the process of aspiration or by seeding of the cancer cells in the port but in this case, we did calculate the Risk of malignancy index (RMI) score and were quite confident that it was a benign cyst.

Paratubal/fimbrial/paraovarian cyst are seen in 4% of women and comprises 10% of all adnexal masses. Paratubal cyst are a remnant of Wolffian duct or Mullerian duct. The cyst occurring from the remnant of Mullerian duct tends to lie close to broad ligament but those from Wolffian duct tend to occur more commonly near the fimbriated end of fallopian tube.^7^

Most of the time, these cysts are small in size around 1 to 8 cm in size and do not cause trouble but sometimes they can be large and lead to complications like cyst rupture, torsion, or intracystic hemorrhage.^1^ They are usually found in the third or fourth decade of life.

For the evaluation of the adnexal cyst, ultrasonography is done which shows “split sign” i.e., there is dissociation of the cyst from the ovary when pushing the probe indicating paratubal/paraovarian cyst but such sign was not present in our case. Demonstration of normal ovary, separate from the cyst is an important finding. However, it was not present in our case.^6^

Though large cyst are challenging to the surgeon due to the limited space and technical difficulty but with careful planning, laparoscopy for a huge cyst is not impractical. Laparoscopic cystectomy of large cyst after decompression of cyst seems to be applicable treatment modality in the background of benign characteristics in tumor marker and imaging.
